# Characterization of Dehydrin protein, CdDHN4-L and CdDHN4-S, and their differential protective roles against abiotic stress in vitro

**DOI:** 10.1186/s12870-018-1511-2

**Published:** 2018-11-26

**Authors:** Aimin Lv, Liantai Su, Xingchen Liu, Qiang Xing, Bingru Huang, Yuan An, Peng Zhou

**Affiliations:** 1 School of Agriculture and Biology, Shanghai Jiao Tong University, Shanghai, 200240 People’s Republic of China; 2 Key Laboratory of Urban Agriculture, Ministry of Agriculture, Shanghai, 201101 People’s Republic of China; 3 Shanghai Chenshan Botanical Garden, Shanghai, 201602 People’s Republic of China; 4 Department of Plant Biology and Pathology, Rutgers, the State University of New Jersey, New Jersey, NJ 08901 USA

**Keywords:** Abiotic stress, Dehydrin, Intrinsic disorder protein, Molecular chaperone, Protein interaction, φ-Segment

## Abstract

**Background:**

Dehydrins play positive roles in regulating plant abiotic stress responses. The objective of this study was to characterize two dehydrin genes, *CdDHN4-L* and *CdDHN4-S*, generated by alternative splicing of *CdDHN4* in bermudagrass.

**Results:**

Overexpression of CdDHN4-L with φ-segment and CdDHN4-S lacking of φ-segment in *Arabidopsis* significantly increased tolerance against abiotic stresses*.* The growth phenotype of *Arabidopsis* exposed to NaCl at 100 mM was better in plants overexpressing CdDHN4-L than those overexpressing CdDHN4-S, as well as better in *E.coli* cells overexpressing CdDHN4-L than those overexpressing CdDHN4-S in 300 and 400 mM NaCl, and under extreme temperature conditions at − 20 °C and 50 °C. The CdDHN4-L had higher disordered characterization on structures than CdDHN4-S at temperatures from 10 to 90 °C. The recovery activities of lactic dehydrogenase (LDH) and alcohol dehydrogenase (ADH) in presence of CdDHN4-L and CdDHN4-S were higher than that of LDH and ADH alone under freeze-thaw damage and heat. Protein-binding and bimolecular fluorescence complementation showed that both proteins could bind to proteins with positive isoelectric point via electrostatic forces.

**Conclusions:**

These results indicate that CdDHN4-L has higher protective ability against abiotic stresses due to its higher flexible unfolded structure and thermostability in comparison with CdDHN4-S. These provided direct evidence of the function of the φ-segment in dehydrins for protecting plants against abiotic stress and to show the electrostatic interaction between dehydrins and client proteins.

**Electronic supplementary material:**

The online version of this article (10.1186/s12870-018-1511-2) contains supplementary material, which is available to authorized users.

## Background

Dehydrins (DHNs) are late embryogenesis abundant (LEA) II proteins, which are thermostable and can maintain their integrity at boiling temperature, playing crucial roles in protecting plants against abiotic stresses such as extreme temperature, drought, and salinity stress [1, 2]. Dehydrin can protect nucleic acids in plant cells during seed maturation and stress responses by interacting electrostatically with vesicles of both zwitterionic and negatively charged phospholipids [3]. Many studies have reported that dehydrins may play a protective function on enzymes or phospholipids as molecular chaperone [4] or molecular shield [5]. Dehydrin ERD10 and ERD14 have chaperone activities with rather wide substrate specificity and interact with phospholipid vesicles through electrostatic forces under cold stress [6], while dehydrin *PpDHNA* and *PpDHNB* from *Physcomitrella patens* provide protection as molecular shields to lactate dehydrogenase (LDH) under osmotic and freezing conditions [7]. KS-type dehydrin (AtHIRD11) can recover the LDH activity denatured by Cu^2+^ in *Arabidopsis* [8]. Hughes and Graether [9] proposed that the dehydrin would stay preferentially localized near the enzyme rather than bind with LDH. Furthermore, there was an extremely weak association between the K-segment of K_2_ and LDH, which was mediated by long-range electrostatic forces. Kovacs et al. [10] argued that the strict distinction between classical chaperone action and molecular shield activity might not be tenable. Although the protective functions of dehydrins to plants exposed to abiotic stress conditions in vivo or *vitro* have been reported [11], the protective mechanism is not completely understood.

Dehydrins are believed to have highly flexible structures because they are composed of charged and polar amino acids [3, 12], and are found to belong to intrinsically disordered proteins (IDPs) [2, 13, 14]. IDPs are natively unstructured proteins that lack defined secondary and tertiary structures [12], which can interact with multiple partners (such as proteins, nucleic acids, membrane and metal ion) in protein interaction networks, and provide important advantages in molecular recognition through transient protein-protein interactions [15]. This type of proteins plays crucial roles in physiological and molecular processes of plants and animals, and works as signaling proteins, transcription factors, or stress response proteins [16–18]. The sequence characteristics of dehydrins possess conserved several segments, which include K-, Y- or S-segments. K-segments (EKKGIMDKIKEKLPG or similar sequence) present in each dehydrin, form an amphipathic helix and are required for binding to anionic phospholipid vesicles [3, 6, 19]. Y-segments, with the core sequence DEYGNP, are located in the N-terminal region of many dehydrins. It has significantly high identity with the nucleotide-binding site of plant and bacterial chaperones [20]. S-segments (LHRSGSSSSSSSEDD) are proposed to affect dehydrin’s nuclear localization [21]. Many dehydrins also contain less conserved φ-segments, which are enriched with polar amino acids, Gly or a combination of Pro and Ala, and present in one or more copies between the K-segments [19, 22]. These enriched polar residues make DHNs flexible [9]. The φ-segments have a structure of random coil, which may allow the φ-segments to bind substantial amounts of water due to interaction of dipolar peptide bonds with water molecules [23, 24]. However, how the presence of φ-segments in DHNs may affect stress protection is not clear.

**Fig. 1:**
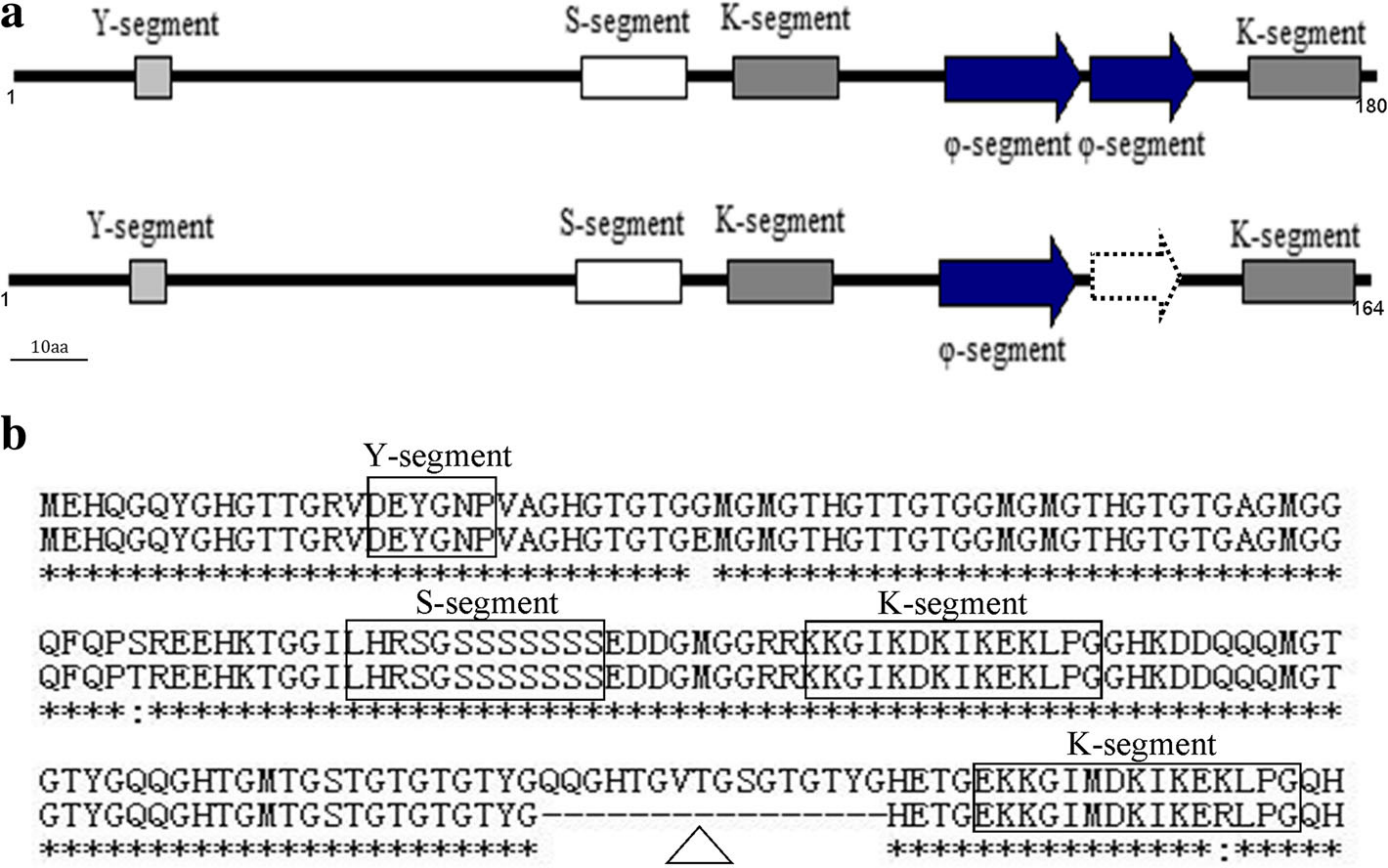
Schematic representation of dehydrin protein. **a** Schematic representation of dehydrin contains domains: top, CdDHN4-L; bottom, CdDHN4-S. φ-segment deletion (white dashed arrows). **b** The amino acids sequence alignment between CdDHN4-L and CdDHN4-S

In our previous studies, two dehydrins, *CdDHN4-L* (accession no. KX243552) and *CdDHN4-S* (accession no. KX243553), were cloned from bermudagrass (*Cynodon dactylon x Cynodon transvaalensis*) ‘Tifway’, which are from RNA alternative splicing with exactly the same genomic DNA sequence [25]. The CdDHN4-L and CdDHN4-S are different in a small protein fragment ^143^QQGHTGVTGSGTGTY^158^G, namely φ-segment (Fig. 1). Due to the binding function of φ-segment to water molecules [23, 24], it was hypothesized that the deletion of φ-segment in CdDHN4-S may make it function differently from CdDHN4-L in plant tolerance to abiotic stresses. Therefore, the objectives of this study were to: 1) compare the structure and functions of the two dehydrin proteins due to a φ-segment deletion; 2) determine the differential mechanisms of the two dehydrin proteins in regulating abiotic stress tolerance (including osmotic, salt, heat or low temperature) in *Arabidopsis*.

## Results

### Expression of CdDHN4-L or CdDHN4-S in plants increased growth rates under abiotic stresses

**Fig. 2:**
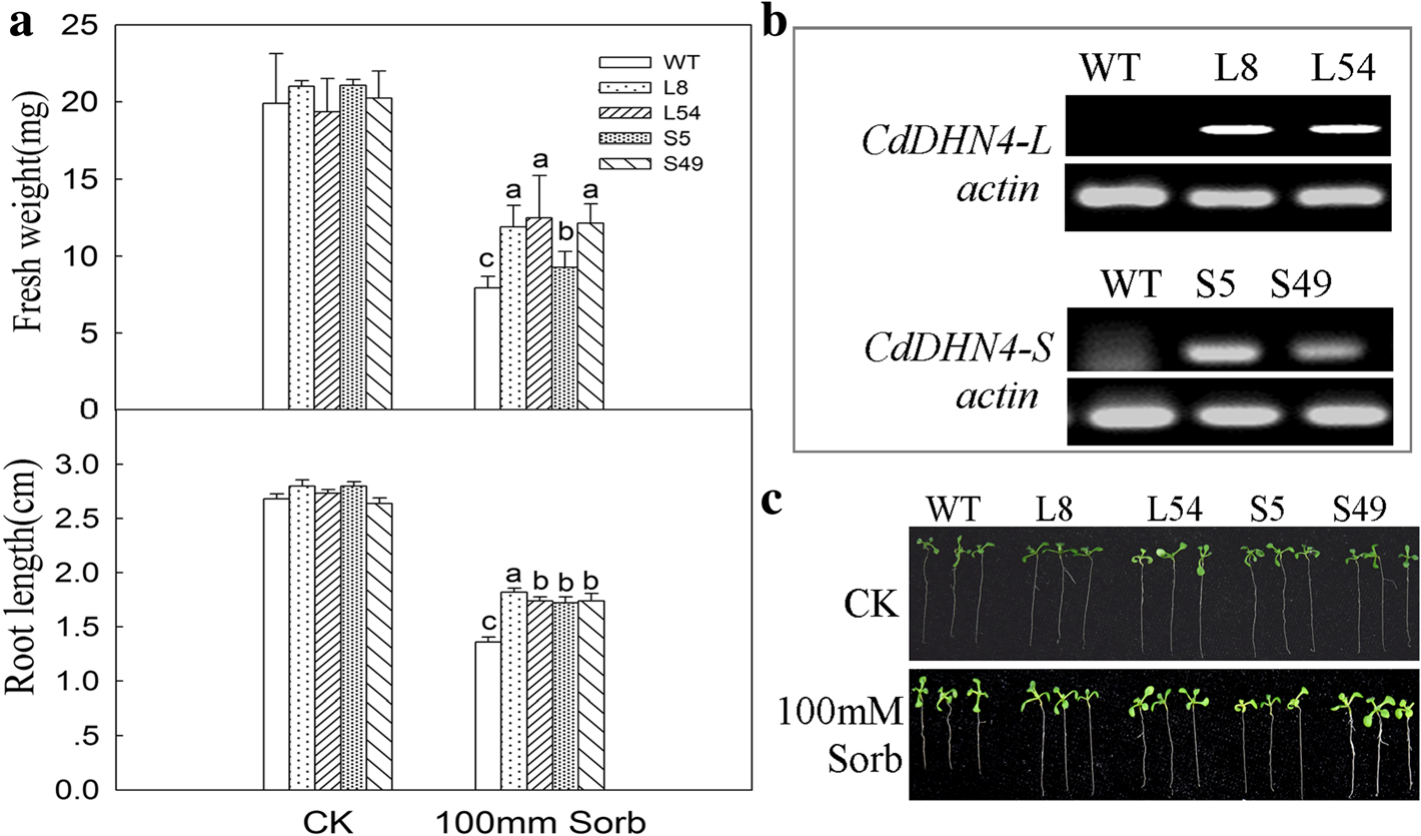
Phenotype of *CdDHN4* transgenic plants under drought stress. **a** Fresh weight and root length of plant under 0 or 100 mM Sorb for 10 days (SE, *n* = 4). **b** The semi-quantitative of CdDHN4-L or CdDHN4-s in 2-week-old wild type and transgenic plants under normal condition. Phenotypes (**c**), plant under 0 or 100 mM Sorb for 10 days (SE, *n* = 4). WT-wild type *Arabidopsis* (Columbia), L8/L54-*Arabidopsis* overexpressing *CdDHN4-L,* S5/S49-*Arabidopsis* overexpressing *CdDHN4-S*. Fresh weight was calculated as the weight of five seedlings. Data are representative of three independent experiments. Different letters show significant differences (*p* < 0.05) as determined by ANOVA analysis

**Fig. 3:**
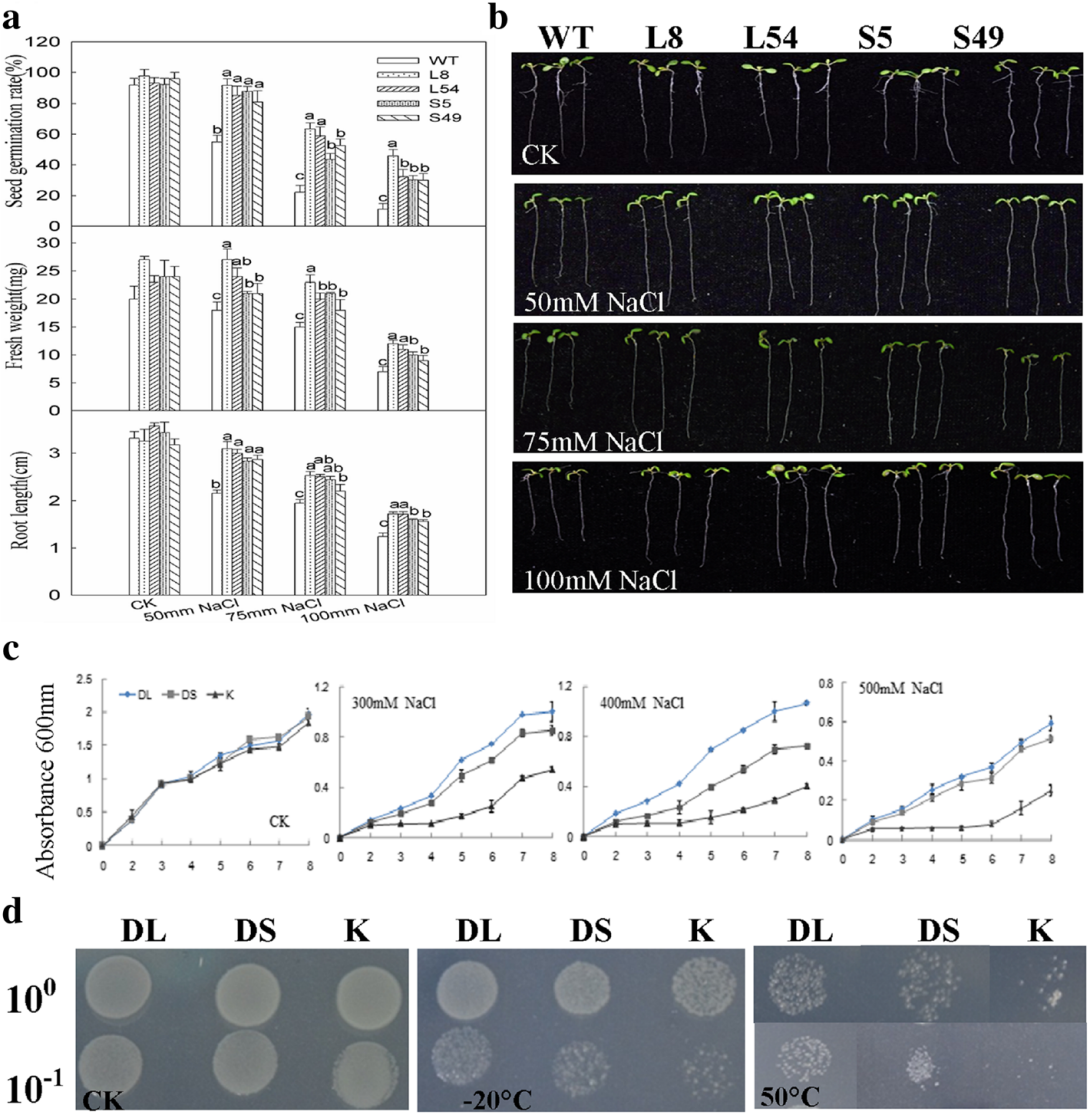
Phenotype of *CdDHN4* transgenic plants under salt stress. Seed germination rate, fresh weight, primary root length (**a**) and phenotypes (**b**) of one-week-old plants under 0, 50, 75, 100 mM NaCl stress. Seed germination rate was measured after 3 days. Growth of IPTG induced *E.coli* cultures producing CdDHN4-L (DL), CdDHN4-S (DS) or pET-21a (K) with control or different stress treatment. *E.coli* Rosetta cells harboring CdDHN4-L, CdDHN4-S or empty vector pET-21a were grown as mentioned above, and diluted to OD_600_ = 0.6. *E.coli* cells (600 μl) were inoculated in 10 mL LB medium and incubated at 30 °C. **c** For salt stress, the LB medium included five concentrations of NaCl (300, 400, and 500 mM). The bacterial suspension was obtained every hour for growth as described previously (Drira et al., 2015). **d** For temperature treatments, IPTG induced cultures (OD_600_ = 0.6) were exposed to − 20 °C for 2 h or 50 °C for 45 min. A 10 μL of each sample diluted by 10-fold was spotted on LB plates, and incubated at 37 °C for 16 h

Transgenic lines with high expression levels of dehydrin genes (Fig. 2b) were used for further investigation. No significant differences were observed in fresh weight, root length (Fig. 2) between wild-type and transgenic *Arabidopsis* seedlings under control treatments. The root length and fresh weight of *Arabidopsis* overexpressing *CdDHN4-L* or *CdDHN4-S* were significantly higher than wild-type at 10 days of 100 mM sorbitol stress (Fig. 2a). In response to salt treatment, the seed germination rate, root length and fresh weight of *Arabidopsis* overexpressing *CdDHN4-L* or *CdDHN4-S* were significantly higher than that of wild-type *Arabidopsis* under 50, 75 and 100 mM NaCl treatment (Fig. 3). *Arabidopsis* overexpressing CdDHN4-L exhibited better growth status than those overexpressing CdDHN4-S under 100 mM NaCl. *E.coli* cells harboring CdDHN4-L exhibited significantly higher growth rate than those harboring CdDHN4-S under NaCl conditions at 300 and 400 mM, and under extreme temperature conditions at − 20 °C and 50 °C (Fig. 3c and d).

### Intrinsic disorder protein character of CdDHN4-L and CdDHN4-S

**Fig. 4:**
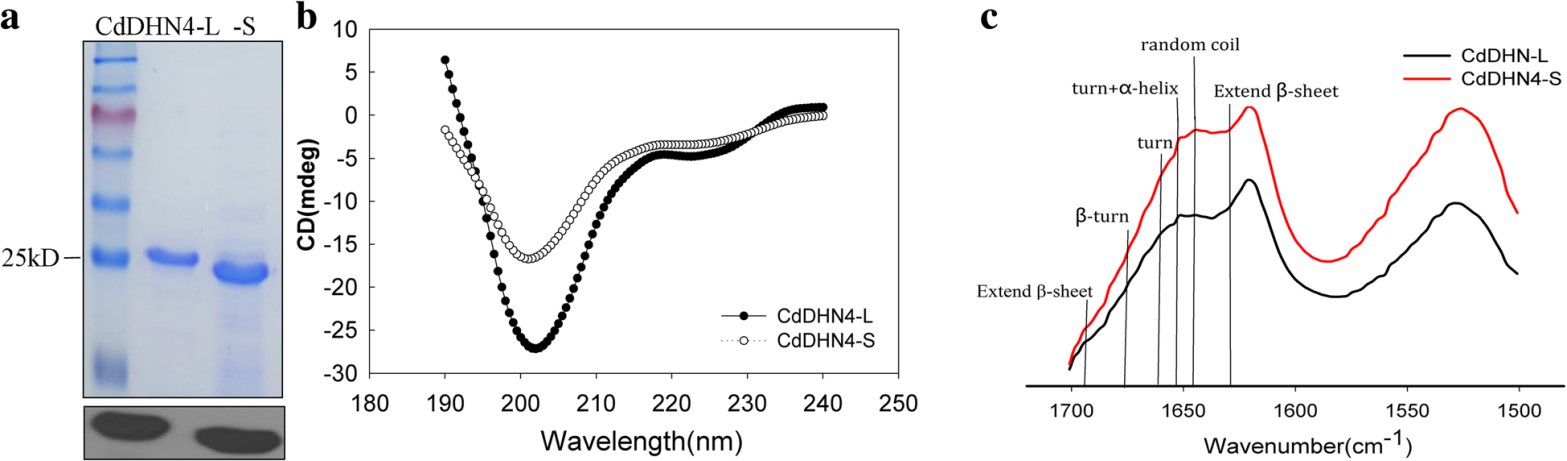
Intrinsically disordered character of CdDHN4-L and CdDHN4-S. **a** Purified protein were detected by SDS-PAGE and stained with Coomassie brilliant blue and after were transferred to PVDF membranes and probed with the anti-K segment of DHNs antibody. **b** CD spectra of CdDHN4-L and -S were recorded at the same protein concentration (0.25 mg mL^− 1^) in deionized water at pH 7.5 and 25 °C. The CD of two proteins difference is not due to differences in protein concentrations or buffer contaminants after protein purification. **c** FTIR analysis of CdDHN4-L and -S at room temperature with (1 mg powder)

The purified CdDHN4-L and CdDHN4-S proteins migrated as larger proteins in the SDS-PAGE analysis, with molecular masses at 25 kDa and 23 kDa for CdDHN4-L and CdDHN4-S, respectively (Fig. 4a). The structure diagnostic analysis showed that CdDHN4-L and CdDHN4-S were hydrolyzed when the ratio of dehydrin protein: trypsin was 1:10, while BSA remained until the ratio was 1:5. The CdDHN4-L and CdDHN4-S proteins were hydrolyzed by low concentration of protease in comparison with BSA (Additional file 1).

Both the CD spectra of the two dehydrin proteins had a large negative peak close to 200 nm at room temperature, which were typical of IDPs (Fig. 4b). The CdDHN4-L and CdDHN4-S were highly disordered proteins, as seen from analyzing the secondary structures of recombinant proteins by CD Spectra software, in which the disordered structure ratio of CdDHN4-L was 64.6%, which was higher than that of CdDHN4-S (61.1%). Other secondary structures such as β-strand and α-helix appeared differently between the two dehydrins.

FTIR gives the amide-I and -II bands between 1,700 and 1,500 cm^− 1^. Both proteins have a shoulder and band at 1640–1645 cm^− 1^, which indicated random coil conformation (Fig. 4c). The second derivative spectra were calculated (Additional file 2). Spectra of CdDHN4-L and CdDHN4-S showed similar profiles: three major bands at 1620, 1633/1634 and 1655 cm^− 1^, and four minor bands at 1656, 1666/1667, 1678 and 1693/1691 cm^− 1^ in amide-I region. The Spectra were lower in CdDHN4-L than CdDHN4-S. The curve-fitting results of the amide-I region showed that CdDHN4-L had 63.2% disordered conformation, 11.1% α-helix and 25.8% β-sheet, while CdDHN4-S had 62.3% disordered conformation, 10.4% α-helix and 23.3% β-sheet (Additional file 2).

### Secondary structures of CdDHN4-L and CdDHN4-S protein at different temperatures

**Fig. 5:**
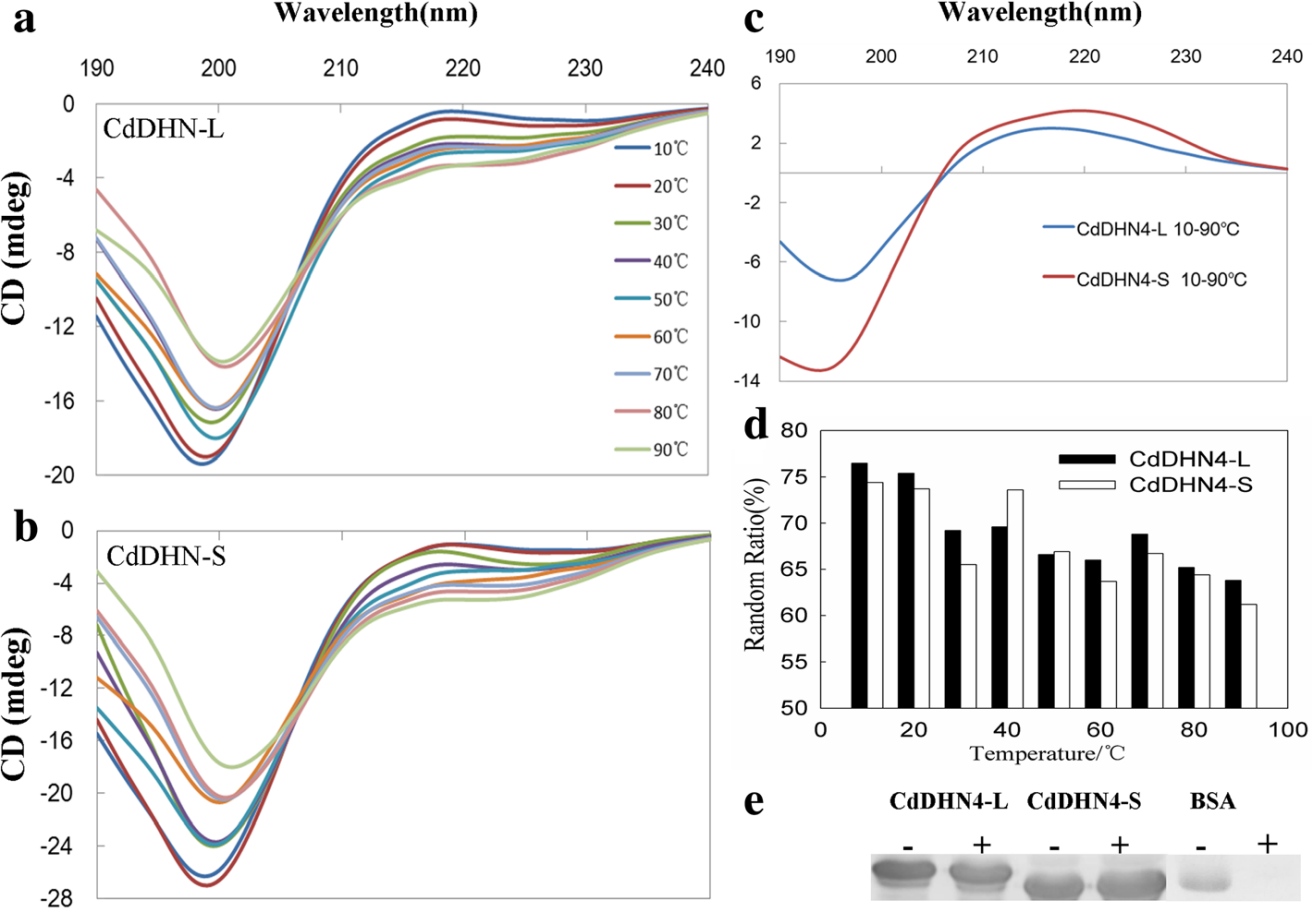
The thermal stability of the CdDHN4 protein. CD spectra of CdDHN4-L (**a**) and CdDHN4-S (**b**) to analyze the effect of increasing temperature on the protein secondary structures. The spectra were measured in 10°Cincrements in deionized water, pH 7.0, from 10 to 90 °C. **c** Difference spectra obtained after subtracting the spectra of both proteins from spectra obtained at 10 and 90 °C. **d** The changes of random ratio in CdDHN4-L or S with increasing temperature. **e** CdDHN4-L and CdDHN4-S and BSA were (+) or were not (−) incubated at 100 °C for 10 min. The samples were sediment with centrifugation 12000 rpm 30 min, and the supernatants were run on SDS-PAGE

The secondary structures of recombinant CdDHN4-L and CdDHN4-S proteins were analyzed by CD spectroscopy in ultrapure water at temperatures from 10 to 90 °C. The spectra of CdDHN4-L and CdDHN4-S proteins had a strong negative band near 200 nm, and a positive shoulder band near 220 nm under different temperatures (Fig. 5a and b). The later band indicated that both proteins contained other secondary structures. There was a balance between disordered structure and the other secondary structure under different temperatures, and the equilibrium point was near 206 nm. Moreover, the CD spectroscopy values between 10 °C and 90 °C were different between the two proteins, in which the largest value (negative bands) of CdDHN4-L and CdDHN4-S occurred at 197 nm and 195 nm, respectively (Fig. 5c). The rate of random coil structures of CdDHN4-L and CdDHN4-S at temperatures from 10 to 90 °C decreased from 76.5 to 61.8% and 74.4 to 63.2%,respectively, while beta structures were increased by 14 and 3%, respectively, and helical structures of CdDHN4-S increased by 8.2%. The random ratios were higher in CdDHN4-L than in CdDHN4-S from 10 to 30 °C and from 60 to 90 °C, while the random ratios in CdDHN4-L were higher or similar to CdDHN4-S at 40–50 °C (Fig. 5d). In addition, the bands of CdDHN4-L and CdDHN4-S proteins were still present at 100 °C for 10 min, but the band of control (BSA) was disappeared (Fig. 5e).

### The protective function of CdDHN4-L and CdDHN4-S in vitro

**Fig. 6:**
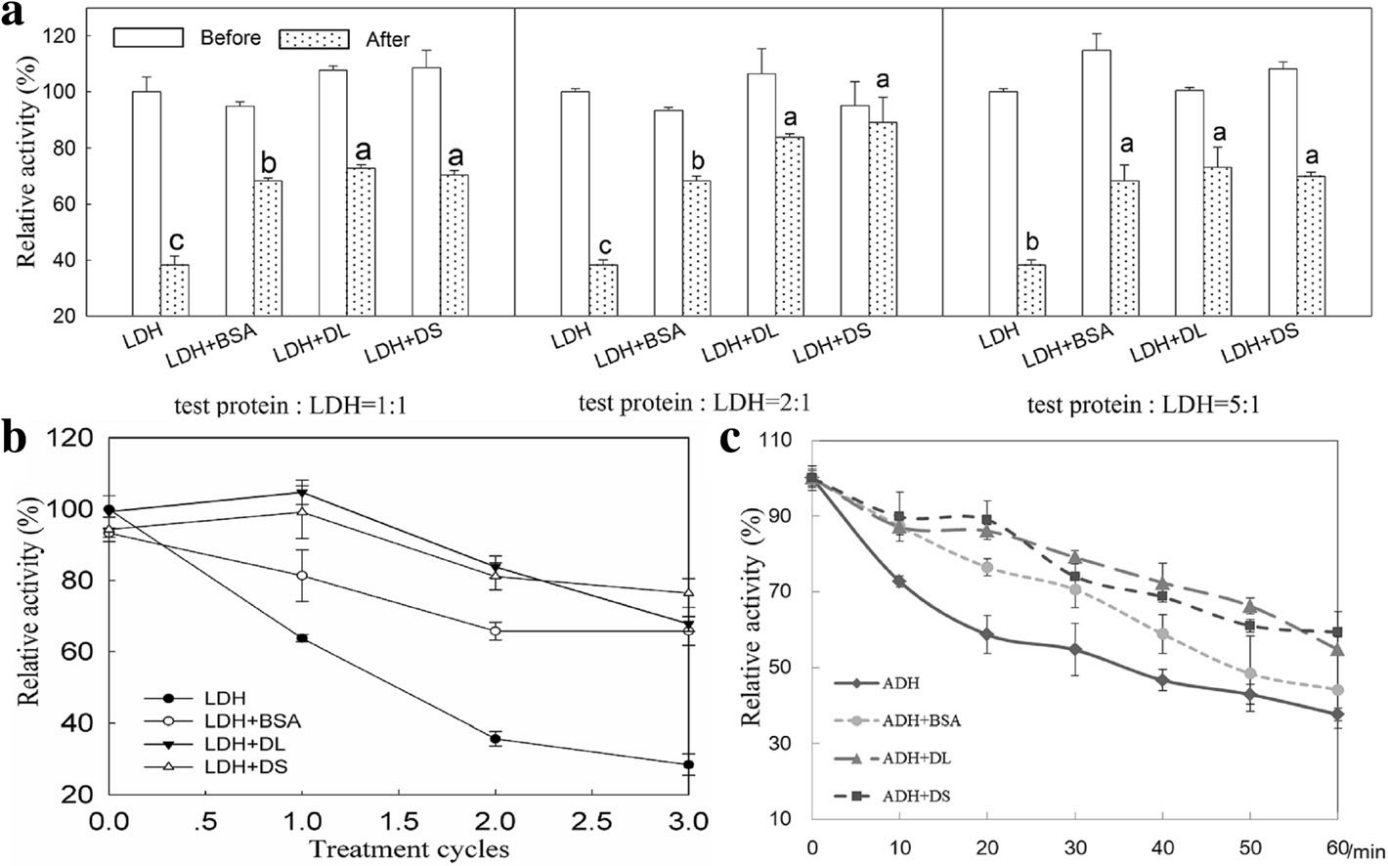
Effect of CdDHN4-L and CdDHN4-S on lactate dehydrogenase (LDH) activity and alcohol dehydrogenase (ADH). **a** CdDHN4-L and CdDHN4-S at different concentrations was added to LDH. Relative enzyme activities are shown as percentage. Before-Activities of non-treated enzymes, After- the reaction solution was incubated 10 min at 50 °C. Values and bars represent means ± SD (three individual experiments). Different letters show significant differences (p < 0.05) as determined by ANOVA analysis among addition with BSA, CdDHN4-L and CdDHN4-S. **b** Cryoprotective activity was assayed with a ratio of 2:1 (test protein: LDH). LDH alone or in present of CdDHN4-L, CdDHN4-S or BSA protein were frozen in − 20 °C and then thawed at room temperature. **c** The heat-induced inactivation of ADH was carried out at 45 °C for 1 h, and enzyme activity was determined every 10 min. Activities of non-treated enzymes are standardized (100%)

Both dehydrins and BSA were potent stabilizers for LDH at 50 °C with different concentration ratios of 1:1, 2:1 and 5:1 (test protein: LDH) (Fig. 6a). The relative activities of LDH incubated with CdDHN4-L or CdDHN4-S at 50 °C were higher than that with BSA at concentration ratios of 1:1 and 2:1 (test protein: LDH), especially when the ratio was 2:1. Similar results were obtained when LDH was placed at freeze(− 20 °C)-thawing conditions (Fig. 6b). The relative activities of LDH were completely recovered to the normal level (0 cycle) in the presence of the two dehydrins at first two freeze-thawing cycles and the activities of LDH were significantly higher in the presence of CdDHN4-L and CdDHN4-S than in BSA at first two freeze-thawing cycles. The relative activities of LDH incubated with CdDHN4-L and -S and BSA at 50 °C or freeze-thawing cycles were significantly higher than that of LDH alone (Fig. 6a and b).

Both dehydrins greatly prevented the loss of ethanol dehydrogenase (ADH) activities during 60 min heating treatments (Fig. 6c). The relative activities of ADH were significantly higher in the presence of CdDHN4-L and CdDHN4-S than in water controls during 60 min heating treatments, and also were significantly higher than that in the presence of BSA during most of heating time.

### Protein-protein interaction

**Fig. 7:**
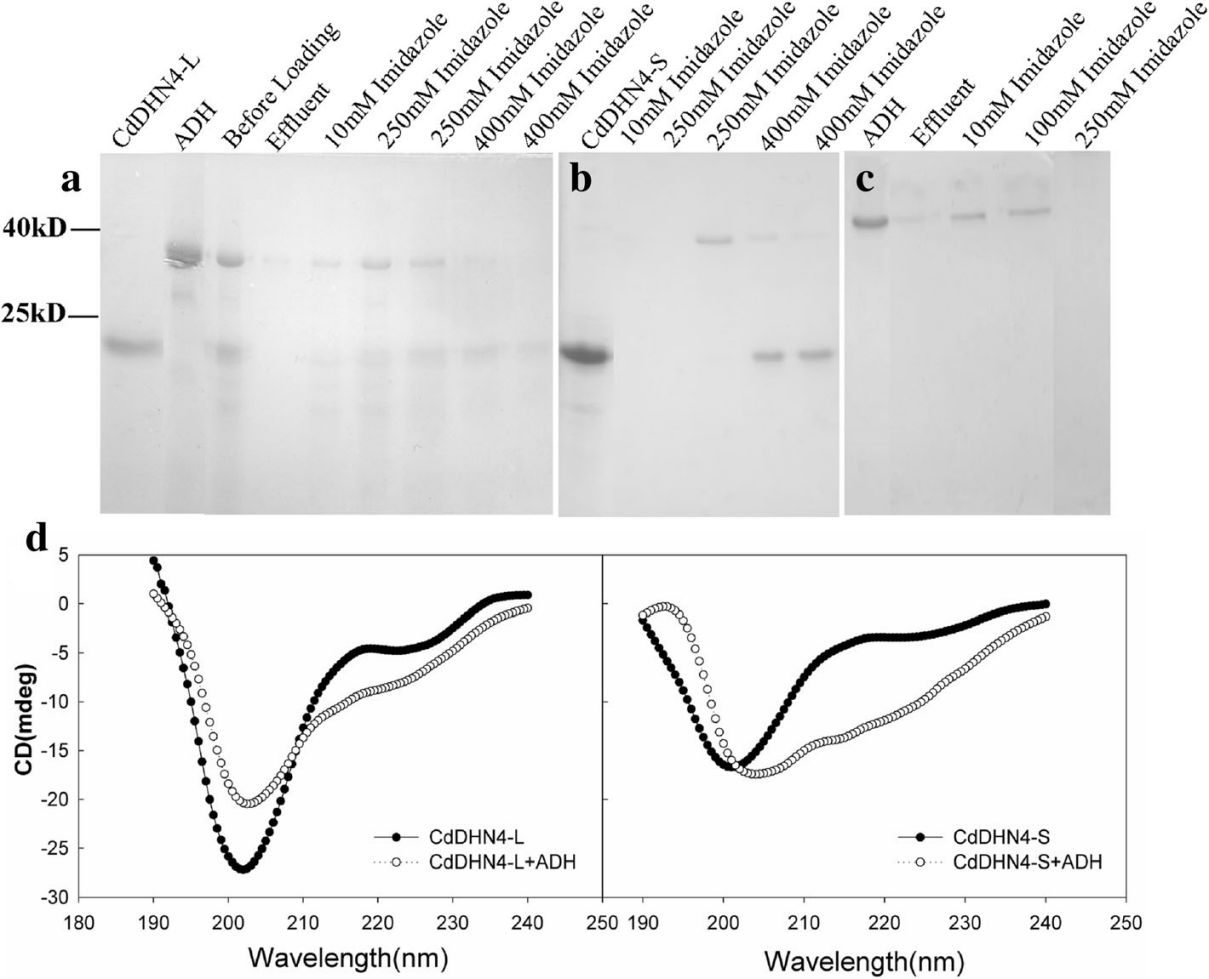
The interaction between ADH and CdDHN4-L, CdDHN4-S in vitro. CdDHN4-L (**a**), CdDHN4-S (**b**) with ADH or ADH alone (**c**) passed through the ProteinIso® Ni-NTA Resin column. Different concentrations of imidazole eluted columns. The effluent was analyzed with SDS-PAGE. **d** CdDHN4-L (left) and CdDHN4-S (right) (0.25 mg mL^− 1^) were analyzed by CD in the presence of ADH (0.125 mg mL^− 1^) at room temperature

**Fig. 8:**
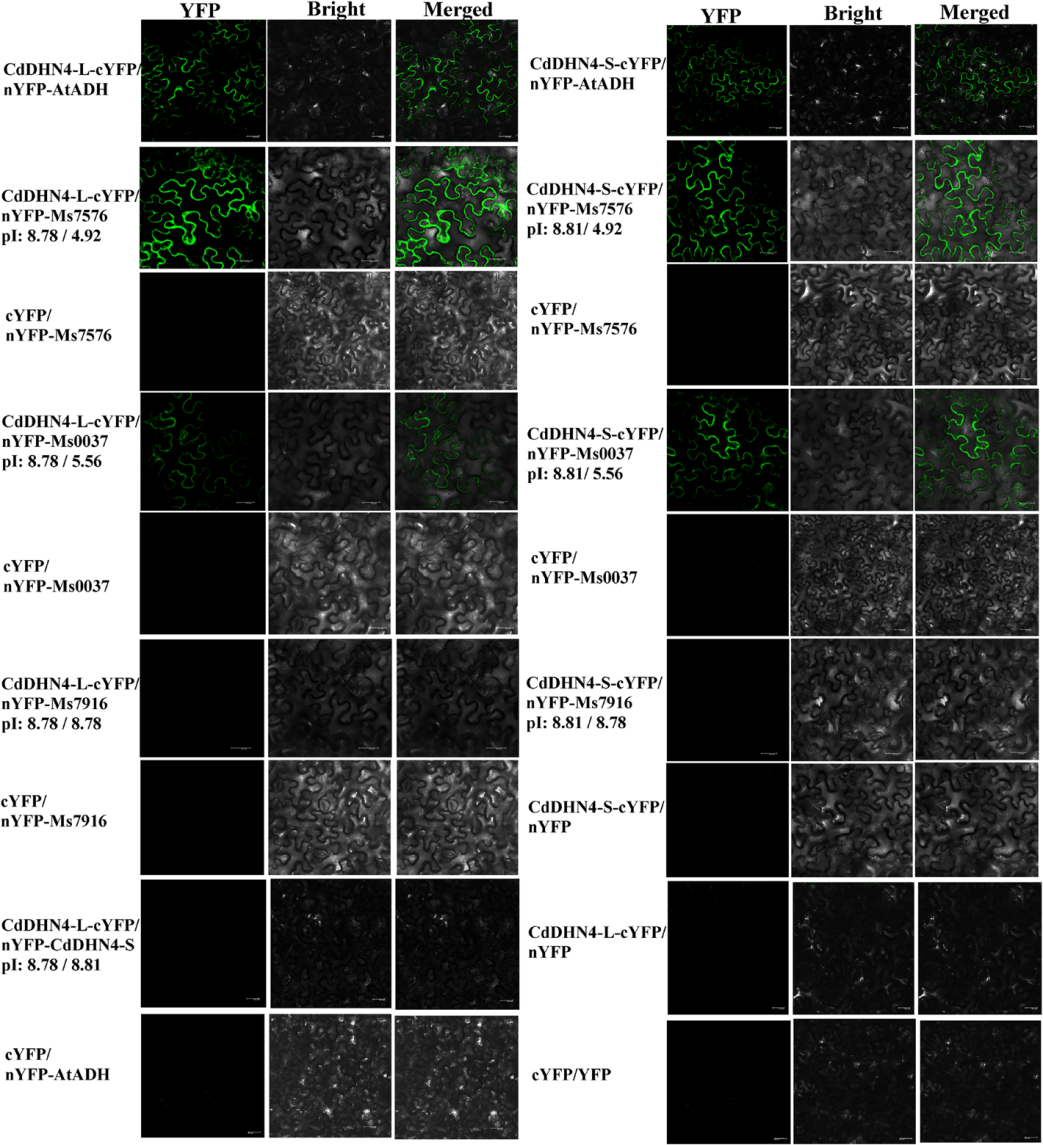
The interaction between CdDHN4-L, CdDHN4-S and other proteins by BiFC analysis. CdDHN4-L and CdDHN4-S were fused with the C-terminal fragment YFP to form CdDHN4-L/S-cYFP. AtADH (*Arabidopsis thaliana*), Ms7576, Ms0037 and Ms7916 were fused with N-terminal fragment of YFP, respectively. cYFP and nYFP represent negative control. Bars, 50 μm

The interactions between two dehydrins and ADH were studied using the two methods of the protein binding and BiFC assay. The analyses of ADH protein binding with CdDHN4-L and CdDHN4-S using ProteinIso® Ni-NTA Resin columns showed that ADH alone was fully eluted by 100 mM imidazole (Fig. 7c), while most of ADH was eluted by 250 and 400 mM imidazole in the presence of CdDHN4-L or CdDHN4-S (Fig. 7a and b). The ADH was not eluted by 10 mM imidazole in the presence of CdDHN4-S, and only a small amount of ADH was eluted by 10 mM imidazole in the presence of CdDHN4-L, but a large amount of ADH was eluted by 10 mM imidazole without CdDHN4-L or CdDHN4-S (Fig. 7c). In addition, YFP fluorescence were detected in tobacco leaves co-transformed the recombinant vectors CdDHN4-L-cYFP and nYFP-AtADH, and vectors CdDHN4-S-cYFP and nYFP-AtADH, but were not detected in the negative control and in vectors CdDHN4-L-cYFP and nYFP-CdDHN4-S (Fig. 8).

The CD analysis showed that large negative peaks near 200 nm were observed after addition of ADH to CdDHN4-L or CdDHN4-S, although the degree of the negative peak of CdDHN4-L was mitigated by the ADH addition, CdDHN4-L and CdDHN4-S still kept the intrinsic disorder protein character in the presence of ADH (Fig. 7d), but the positive peak of CdDHN4-S near 220 nm was lost after addition of ADH (Fig. 7d). In the presence of ADH, the beta conformation proportion of CdDHN4-L and CdDHN4-S increased from 23.1 to 33% and from 27.3 to 45.8%, respectively, while the random coil proportion decreased from 64.6 to 55%, and from 61.4 to 51%, respectively.

### The protein-protein interaction via electrostatic interaction

The isoelectric point (pI) of AtADH protein (*Arabidopsis thaliana*, AT1G77120) was 6.19, while the pIs of CdDHN4-L and CdDHN4-S were 8.87 and 8.81, respectively. The difference of pIs between CdDHN4-L or CdDHN4-S and AtADH, and between CdDHN4-L and CdDHN4-S might be related to their protein interactions mentioned in above paragraph. To confirm this prediction, the interactive relationships between CdDHN4-L or CdDHN4-S and other three proteins with different pIs were analyzed by BiFC assay. The results were shown in Fig. 8. The YFP fluorescence were detected in the vectors CdDHN4-L/S-cYFP and nYFP-Ms7576 with pI 8.78/8.81 and 4.92, respectively, and in the vectors CdDHN4-L/S-cYFP and nYFP-Ms0037 with pI 8.78/8.81 and 5.56, but no YFP fluorescence was detected in the vectors CdDHN4-L/S-cYFP and nYFP-Ms7916 with pI 8.78/8.81 and 8.78, and in other vectors. The YFP fluorescence intensities decreased with the reduction of pI difference between the two interactive proteins.

## Discussion

In the present study, overexpression of *CdDHN4-L* and *CdDHN4-S* cloned from bermudagrass enhanced tolerance against drought and salt stresses in transgenic *Arabidopsis* and tolerance against salinity, low or high temperature stresses in transgenic *E.coli* cells, suggesting that these two dehydrins play positive roles in stress defense, similar to reported by others in plants and bacteria [26–28]. The novelty of this study is reflected by the differential effects of CdDHN4-L and CdDHN4-S. The growth rate of *Arabidopsis* and *E.coli* cells overexpressing CdDHN4-L exposed to NaCl at 100 mM (*Arabidopsis*) and NaCl at 300–400 mM and temperature at − 20 °C and 50 °C(*E.coli* cells) were higher than that overexpressing CdDHN4-S. The stability of the two dehydrin proteins under abiotic stressed conditions may affect their function on tolerance of plants against stresses. In our study, the disorder structure proportion of CdDHN4-L were higher than that of CdDHN4-S from 10 to 30 °C and from 60 to 90 °C, but proportion of CdDHN4-S was higher at 40 °C in comparison with CdDHN4-L. These results indicated that CdDHN4-L was more stable than CdDHN4-S under moderate and severe stress conditions.

The K, Y and S-segments are major domains of dehydrin proteins, which determine the function of dehydrin protein. In addition, most of dehydrins have another domain, φ-segments [19]. The φ-segments are seldom studied and no direct evidence showed its function in dehydrin proteins because of its less conserved trait [12, 29]. Recently, Rosales et al. [22] first compared two spliced dehydrin variants, the spliced DHN1a_s and the unspliced DHN1a_u which lacked φ- and K-segments, and found that DHN1a_s had a potent cryoprotective effects on LDH activity and malate dehydrogenase activity against dehydration and partially inhibited *B.cinerea* growth, and concluded that φ- and K-segments played role in DHNs function in plant response to abiotic stress. However, until then there is no direct evidence showing the function of φ-segments. In the present study, CdDHN4-S with the φ-segment deletion exhibited differential effects from CdDHN4-L containing the φ-segment, which first provided the evidence of the positive roles of φ-segment in protecting plants against abiotic stress.

Dehydrins are intrinsic disorder proteins with flexible secondary structure [30]. In the present study, the CdDHN4-L and CdDHN4-S expressed typical dehydrin characteristics in the following aspects: 1) a low mobility, as seen from SDS–PAGE and western blot analysis [10, 22]. The low mobility maybe attributed to their phosphorylation sites and high hydrophilcity [31]; 2) the higher sensitivity to proteases K and trypsin than BSA, which are attributed to their polypeptide chain that determines dehydrins to be more accessible to proteases than that of globular proteins [32]; 3) the random coil proportion of two dehydrins accounted for over 60% of total secondary structure under temperatures from 10 to 90 °C by CD analysis, and over 62.3% of unstructured ratio by FTIR analysis. The CdDHN4-L had higher random coil proportion than CdDHN4-S in both two analyses. These results demonstrated that CdDHN4-L and CdDHN4-S, especially CdDHN4-L, had highly flexible unfolded structure and thermal stability, exhibiting the properties as IDPs.

Disordered dehydrins have been shown to reduce radical reactive oxygen species from transition metal [2], and protect enzymes from free-thaw damage or heat denaturation [29, 32]. In this study, the recovery activities of LDH and ADH in presence of CdDHN4-L and CdDHN4-S were greatly higher than that of LDH and ADH alone under free-thaw damage and heat stresses, indicating that the two dehydrins have strong ability of protecting enzymes, which can be attributed to their flexible unfolded structures under abiotic stress conditions [33, 34]. Several studies have shown that dehydrins are able to bind to membranes [3, 35], water [36] and metal ions [37, 38]. In this study, CdDHN4-L and CdDHN4-S prevented ADH elution under lower concentration of imidazole, but the ADH alone was easily eluted under lower concentration of imidazole, indicating that CdDHN4-L and CdDHN4-S were able to bind to ADH protein. The YFP fluorescence observed in vectors of CdDHN4-L/S-cYFP and nYFP-AtADH further confirmed the direct interaction between dehydrins and ADH.

Many evidences show that IDPs always share properties of a flexible conformation, a high proportion of disorder-promoting residues, and a lack of stable structure or only some residual secondary structure when dehydrins scatter in solution or bind with other proteins, metal ions or lipid vesicles [39]. For example, trifluoroacetic acid and slow drying usually induce dehydrins to increase their α-helix [40, 41], while dehydrins binding to lipid vesicles always accompany with an increase in β-sheet [42, 43]. The disorder degrees of CdDHN4-L and CdDHN4-S binding to ADH were lower than that of dehydrin itself, and these conformation changes maybe attribute to the interaction between CdDHN4 and ADH [2]. In addition, β-sheet contents in CdDHN4-L and CdDHN4-S were increased in the presence of ADH. The binding plasticity and self-conformation change allow dehydrins to interact with multiple partners, which are critical for their function [15]. Comparing with CdDHN4-L, CdDHN4-S had different refolding structure in which the positive peak near 220 nm disappeared with addition of ADH, which may be attributed to the φ-segment deletion. Furthermore, the lower flexible unfolded structure and thermal stability in CdDHN4-S may also be attributed to its φ-segment deletion compared with CdDHN4-L. A further study should be conducted to investigate the effect of φ-segment deletion on the structure and function of CdDHN4-L.

The typical cytoplasmic proteins always have a negatively charged surface at physiological pH [44], and the numerous natively unfolded proteins change their conformations after incubation with other molecules such as proteins and ions or chemicals due to their negative charged surface [14]. The dehydrins are unfolded proteins, which have been reported to protective plants exposed to abiotic stresses by molecular chaperone [45]. In the present study, there were strong binding activities between CdDHN4-L or CdDHN4-S with ADH, which increased the activities of ADH and LDH under free-thaw and heat stress. Meanwhile the secondary structure of CdDHN4-L and CdDHN4-S were still kept well under different temperature conditions and in the presence of ADH although the secondary structures had slightly changed, indicating that the two dehydrins played a molecular chaperone function to ADH [5, 46].

The classical chaperone acts to prevent aggregation of unfolded polypeptides and assists in the correct refolding of chaperone-bound denatured polypeptides through electrostatic forces, For example, Eriksson et al. [3] reported that Lti30 (dehydrin) interacted electrostatically with vesicles of both zwitterionic (phosphatidyl choline) and negatively charged phospholipids (phosphatidyl glycerol, phosphatidyl serine, and phosphatidic acid) with a stronger binding to membranes with high negative surface potential. At the present, the molecular chaperone via electrostatic forces is speculated by pI of chaperone and client proteins, and no direct evidence is reported. In the present study, The YFP fluorescence, reflecting binding activities between CdDHN4-L/S and other client proteins via BiFC assay, were detected in the vectors of CdDHN4-L/S-cYFP and nYFP-Ms7576, or nYFP-Ms0037, or nYFP-AtADH, respectively, but no YFP fluorescence were detected in the vectors of CdDHN4-L/S-cYFP and nYFP-Ms7916, as well as CdDHN4-L-cYFP and nYFP-CdDHN4-S. The YFP fluorescence intensities decreased with the reduction of pI difference between the two binding proteins, indicating that the two dehydin proteins were bounded with the first three tested proteins (Ms7576, Ms0037 and AtADH) via electrostatic forces, and the binding ability was positive to the pI difference between the two binding proteins. These results first provided the evidence of molecular chaperone function of dehydrins via electrostatic forces.

## Conclusions

This study demonstrated that CdDHN4-L and CdDHN4-S cloned from bermudagrass were unfolded proteins and overexpression of the two dehydrins enhanced tolerance to drought and salt stress in transgenic *Arabidopsis*, and to salt, extreme temperatures stress in transgenic *E.coli* cells. The tolerance against stresses of CdDHN4-L with the φ-segment was greater than CdDHN4-S lacking of the φ-segment, which was corresponded with the more stability of the unfolded structure proteins under unfavorable temperature. The φ-segment could account for the differential effects between CdDHN4-S and CdDHN4-L. The strong binding activities occurred between CdDHN4-L or CdDHN4-S with Ms7576, Ms0037 and AtADH via close-range electrostatic forces, and the binding abilities were positive to their pI difference between the two binding proteins. The protective roles of CdDHN4-L and CdDHN4-S on plants against abiotic stresses could be due to the direct protein-protein interaction driving by electrostatic forces. In future studies, we will further evaluate the difference between CdDHN4-L and CdDHN4-S on their structure and function under abiotic stressed conditions by homologous transforming the two dehydrin genes into bermudagrass, and further understand the function of φ-segment in dehydrins for protecting plants against abiotic stress,as well as its influence on protein-protein interaction.

## Methods

### Abiotic stress in *Arabidopsis*

Two dehydrins, *CdDHN4-L* and *CdDHN4-S* have been obtained from bermudagrass (*Cynodondactylon* L.) ‘Tifway’. The sequence of *CdDHN4* without the terminating codon was amplified and inserted into the BamHI/SpeI sites of PHB-CFP to generate the construct PHB-*CdDHN4*-CFP. After sequencing, the constructs were introduced into *Agrobacterium tumefaciens* strain GV3101, and transformed into Arabidopsis (Ecotype Col-0, from Lian’s Lab) by the floral dip method [47]. Seeds of T_3_ transgenic *Arabidopsis* plants overexpressing *CdDHN4-L* or *CdDHN4-S* (Fig. 2b) and the wild type (Columbia) were placed on petri dishes with MS medium and seedlings were grown for two weeks in the petri dishes. For drought stress, the seeds were grown in the MS medium with 0 or 100 mM Sorbitol. Primary root length, fresh weight of whole plant in leaves were measured after 10 days. For salt stress, the transgenic and wild type plants were grown in the MS medium supplemented with 0, 50, 75 and 100 mM NaCl for 7 days. Seed germination rate, primary root length and fresh weight were measured to evaluate effects of salinity stress. All of the experiments were repeated three times.

Total RNA were isolated from plant leaves using the TransZol Up Plus RNA Kit. First-strand cDNA was synthesized using TransScript One-Step gDNA Removal and cDNA Synthesis SuperMix (TransGen Biotech).The semi-quantitative RT-PCR were conducted as follows: 95 °C for 5 min, then 26 cycles of 95 °C for 30 s, 58 °C for 30 s, and 72 °C for 20 s for both *CdDHN4* and *actin*.

### Protein extraction and purification

A recombinant expression of CdDHN4-L and CdDHN4-S proteins (Additional file 3) in prokaryote was performed according to a method previous reported by Hara et al. [2]. In brief, the open reading frames of CdDHN4-L and CdDHN4-S were expressed by the pET-21a *E.coli* expression system (Novagen, WI, USA). These recombinant proteins have His-tag sequences at the C-terminus. The *E.coli* strain Rosetta (DE3) was used as the host. The bacterial stain was pre-cultured at 37 °C, and 1 mM (IPTG) isopropyl-β-dthiogalactopyranoside was added to the culture. After incubation for 6 h at 30-°C, bacterial cells (300 mL of culture) were centrifuged at 12000 *g* for 20 min. Bacterial thallus were lysed by sonication in 1/10 PBS (pH 7.4) and 1 mM phenylmethylsulfonyl fluoride. The lysate was centrifuged at 12000 *g* for 30 min at 4 °C. The CdDHN4-L and CdDHN4-S proteins were purified subsequently by a *ProteinIso®* Ni-NTA Resin (5 mL, TRANSGEN BIOTECH, Beijing, China) immobilizing Ni^2+^ and then were eluted by different concentrations of imidazole. Samples were desalted using an ultrafiltration tube (Millipore Amicon® Ultra). The CdDHN4-L and CdDHN4-S was stored as a water solution (1–2 mg mL^− 1^) at − 80 °C until used, respectively. The recombinant proteins were identified by the sodium dodecyl sulfate-polyacrylamide gel electrophoresis (SDS-PAGE) gel stained with Coomassie Brilliant Blue and western blot with Anti-dehydrin (AS07206, Agrisera, Sweden).

### Circular dichroism spectroscopy

Proteins CdDHN4-L and CdDHN4-S were analyzed with a Circular dichroism (CD) spectropolarimeter (J-815, JASCO, Japan). Protein samples (0.25 mg mL^− 1^) were combined in water solution (pH 7.0) with a 0.1 cm optical path length cell at room temperature. The scan was performed from 190 nm to 250 nm. The scan speed, cell length, and band width were 50 nm min^− 1^, 1 mm, and 2 nm, respectively. Then the secondary structures of both proteins at various temperatures were analyzed at a range of temperatures from 10 to 90 °C.

CdDHN4-L and CdDHN4-S (0.25 mg mL^− 1^) were also subjected to CD spectropolarimeter in the presence of alcohol dehydrogenase (0.125 mg mL^− 1^) at room temperature.

### Fourier transform infrared spectroscopy (FTIR) analysis

FTIR spectroscopy was performed as described by shih et al. [14] with slight modification. Purified recombinant CdDHN4-L and -S proteins were dried in Cryogenic dryer to be solid. The dried protein samples (1 mg) were poured on circular plates (ATR). FTIR spectra in the range between 4000 and 525 cm^− 1^ were recorded at room temperature, with a spectral resolution of 4 cm^− 1^, on an IR/Nicolet 6700 spectrometer (USA).

The secondary structure of two proteins were analyzed at the spectral region between 1700 and 1500 cm^− 1^ according to shih et al. [14]. Secondary structure components were derived from the second derivative spectra of the amide-I bands according to Gaussin FTIR (Software, PeakFit).

### Intrinsic disorder protein analysis

The resistance to heat induced aggregation was analyzed with SDS-PAGE. CdDHN4-L and CdDHN4-S (0.25 mg mL ^− 1^) of and BSA (as a control, its isoelectric point is 8.6) were treated at 100 °C for 10 min and centrifuged at 12,000 *g* for 20 min at room temperature. The supernatants were analyzed with a 12.5% SDS-PAGE gel.

Protease sensitivity of CdDHN4-L and CdDHN4-S were tested with two proteases: proteinase K (broad specificity) and trypsin (narrow specificity). Protein samples (0.5 mg mL^− 1^) were treated with different ratios of protease for 30 s. The reactions were stopped by adding 2*loading buffer and heated at 100 °C for 5 min. Then samples were identified by SDS-PAGE.

### Thermal and freeze-thawing inactivation of lactate dehydrogenase

Lactate dehydrogenase (LDH, from rabbit muscle, Sigma, USA) activity was determined in triplicates using aliquots of 5 μL in 200 μL of 100 mm NaH_2_PO_4_ buffer (pH 6.0), 2 mm pyruvate, 100 mm NADH. The decrease in A_340_ was monitored in 96-well microplate with a Microplate reader (Synergy|^2^, BioTek, USA). Enzymatic activity was followed at room temperature by recording the decrease in 340 nm for 5–6 min. Heat inactivation of LDH was assayed. A mixture of 60 μL containing 10 μg mL^− 1^ LDH and the indicated amounts CdDHN4-L, CdDHN4-S or BSA protein were incubated 10 min at 50 °C. Samples measuring 20 μL were assayed for LDH activity in a final volume of 200 μL. Cryoprotective activity was assayed with a mixture of 60 μL containing 10 μg mL^− 1^ LDH and the same amounts of CdDHN4-L, CdDHN4-S or BSA protein were frozen at − 20 °C and then thawed at room temperatures. Enzyme activities were determined every 10 min until 3 freeze-thawing.

### Thermal inactivation of alcohol dehydrogenase

The heat-induced inactivation of yeast alcohol dehydrogenase (ADH) was carried out at 45 °C for 1 h. ADH (1 mg mL^− 1^) was mixed with same concentration of CdDHN4-L, CdDHN4-S and BSA (as a control) in 100 mM NaH_2_PO_4_ and 0–250 mM NaCl, pH 8.8). The samples were incubated on ice for 5 min before assay. Then, the reaction solution was placed into a 45 °C water bath, and enzyme activity was determined every 10 min. The enzyme activity was measured with 1.25 mM ethanol and 2 mM NAD^+^ (100 mM NaH_2_PO_4_, pH 7.5) by adding 20 μL (to 200 μL) of reaction solution. The increase in NADH A_340_ was followed at room temperature with microplate reader.

### Protein-binding assay

The interaction between protein (such as ADH) and CdDHN4-L or CdDHN4-S was analyzed by a *ProteinIso®* Ni-NTA Resin in vitro. ADH samples were prepared in the presence and absence of purified CdDHN4-L or CdDHN4-S and passed through the *ProteinIso®* Ni-NTA Resin column. Then, different concentrations of imidazole eluted columns. The amount of the protein in the flow through is characteristic of the strength of the interaction. The effluent was analyzed with 12.5% SDS-PAGE.

For Bimolecular fluorescence complementation (BiFC) analysis, the vectors pXY106-nYFP and pXY104-cYFP supplied by Yang Hongquan’s lab were used. Then the full-length open reading frame of *CdDHN4-L*, *CdDHN4-S* and *AtADH* gene (*Arabidopsis thaliana,* Gene ID in NCBI: 844047) were cloned into the expression vectors, respectively. Subsequently, CdDHN4-L-cYFP, CdDHN4-S-cYFP, ADH-nYFP and CdDHN4-S-nYFP were transformed into GV3101 and transiently co-transformed into 5-weeks old tobacco leaves (*N.benthamiana*). Yellow fluorescent protein (YFP) signals were detected by confocal microscopy (Leica Microsystems, Wetzlar, Germany) after 48–72 h in darkness conditions.

Multiple proteins used in BiFC analysis were obtained and kept in our lab (data not shown here). The isoelectric point (pI) of the protein Ms7576, Ms0037 and Ms7916 were 4.92, 5.56 or 8.78, respectively. These protein molecular weights were 27.3, 24.8 or 20.8 kDa, respectively. All molecular weight and pI of the proteins were predicted by DNAMAN software, based on amino acid sequences. All primers used in this study showed in Additional file 4.

### Data analysis

The data of root length, and spectral absorbance were subjected to the analysis of variance and treatment means were separated using the least significance test (LSD) at a probability of 0.05. The characteristics of protein primary sequences, including pI values, net charges and molecular weight were analyzed by ExPASy (http://web.expasy.org/protparam).

## Additional files

Additional file 1: BSA, CdDHN4-L and CdDHN4-S were treated with Trypsin and proteinase K. (DOCX 152 kb)

Additional file 2: Band positions and individual contributions by various secondary structures of CdDHN4-L and CdDHN4-S proteins determined by curve fitting of the composite amide-I band of FTIR spectra. (DOCX 15 kb)

Additional file 3: Analysis of protein extracts from IPTG induced *E.coli* Rosetta (DE3) expressing the recombinant CdDHN4-L and CdDHN4-S. (DOCX 514 kb)

Additional file 4: Primer sets used in this study. (DOCX 17 kb)

## Abbreviations

ADH: Alcohol dehydrogenase
At: *Arabidopsis thaliana*
BiFC: Bimolecular fluorescence complementation
BSA: Albumin from bovine serum
CD: Circular dichroism
DHN: Dehydrin
FTIR: Fourier transform infrared spectroscopy
IDPs: Intrinsically disordered proteins
IPTG: Isopropyl β-D-Thiogalactoside
LDH: Lactic dehydrogenase
LEA: Late embryogenesis abundant
LSD: Least significance test
MS: Murashige and Skoog medium
PAGE: Polyacrylamide gel electrophoresis
pI: Isoelectric point
Sorb: Sorbitol
WT: Wild type
YFP: Yellow fluorescence protein
