# Supplementary material for: Characterization of Dehydrin protein, CdDHN4-L and CdDHN4-S, and their differential protective roles against abiotic stress in vitro
Source: BMC Plant Biol. 2018 Nov 26;18:299. doi: 10.1186/s12870-018-1511-2 (PMC6258397; doi:10.1186/s12870-018-1511-2)
Supplement: Supplementary file 1 — BSA, CdDHN4-L and CdDHN4-S were treated with Trypsin and proteinase K. (DOCX 152 kb) [file 12870_2018_1511_MOESM1_ESM.docx]

**Additional file 1:**


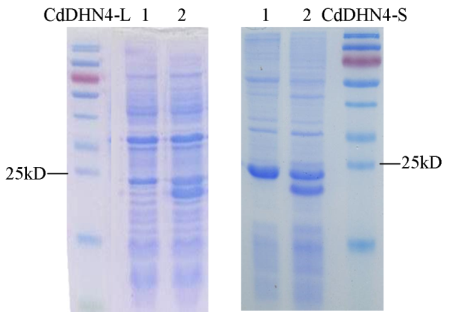


**Additional file 1** Analysis of protein extracts from IPTG induced *E.coli* Rosetta (DE3) expressing the the recombinant CdDHN4-L and CdDHN4-S. Unpurified extracts were separated by SDS–PAGE (12.5% polyacrylamide) and stained with Coomassie brilliant blue, 1-no IPTG, and 2-after added IPTG 3h.
