# Supplementary material for: Characterization of Dehydrin protein, CdDHN4-L and CdDHN4-S, and their differential protective roles against abiotic stress in vitro
Source: BMC Plant Biol. 2018 Nov 26;18:299. doi: 10.1186/s12870-018-1511-2 (PMC6258397; doi:10.1186/s12870-018-1511-2)
Supplement: Supplementary file 2 — Band positions and individual contributions by various secondary structures of CdDHN4-L and CdDHN4-S proteins determined by curve fitting of the composite amide-I band of FTIR spectra. (DOCX 15 kb) [file 12870_2018_1511_MOESM2_ESM.docx]

**Additional file 2:**

**Table 1 Primer sets used in this study**

| Name | 5’- 3’ sequence |
| --- | --- |
| CdDHN4-F | CGGGATCCATGGAGCACCAGGGACAGTAC |
| CdDHN4-R | CCCCCGGGATGGAGCACCAGGGACAGTAC |
| CdDHN4-R | AACTGCAGTTAGTGCTGGCCGGGGAGCTT-3 |
| AtADH-F | CGGGATCCATGTCTACCACCGGAC |
| AtADH-R | GCGTCGACTCAAGCACCCATGGTG |
| Ms7916-F | CGGGATCCATGACTATTGCTAGGGCTCGT |
| Ms7916-R | AACTGCAGTCAATTCTTTTTGTTCATGTTCCTC |
| Ms7576-F | CGGGATCCATGAGTCAAGAACAGCCACAGA |
| Ms7576-R | GCGTCGACTCACCCGCTCTTCGTGTTCTGG |
| Ms0037-F | CGGGATCCATGGCTGAGGAGAATCAGAACA |
| Ms0037-R | GCGTCGACTCAATGAGAAGTAGTCTCATCCTTG |

Note: The underscore represents the restriction enzyme site..
