# Supplementary material for: Characterization of Dehydrin protein, CdDHN4-L and CdDHN4-S, and their differential protective roles against abiotic stress in vitro
Source: BMC Plant Biol. 2018 Nov 26;18:299. doi: 10.1186/s12870-018-1511-2 (PMC6258397; doi:10.1186/s12870-018-1511-2)
Supplement: Supplementary file 3 — Analysis of protein extracts from IPTG induced E.coli Rosetta (DE3) expressing the recombinant CdDHN4-L and CdDHN4-S. (DOCX 514 kb) [file 12870_2018_1511_MOESM3_ESM.docx]

**Additional file 3:**


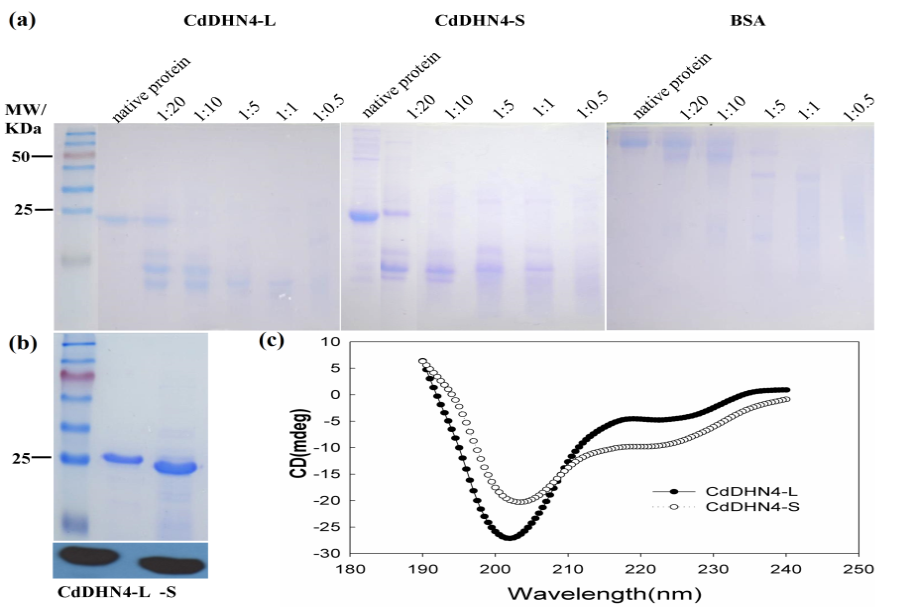


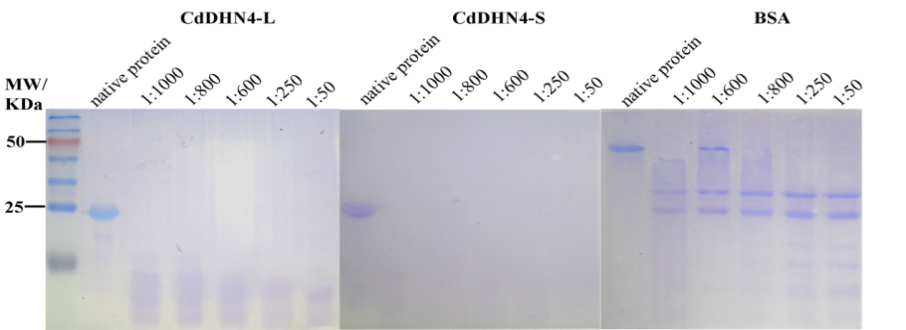


**(b)**

**Additional file 3** (a) Limited proteolysis of BSA, CdDHN4-L and CdDHN4-S with trypsin. The ratio of proteinase concentration (mg mL^-1^) was varied from 1:20 (dehydrin protein: trypsin) to 1:0.5 relative to substrate concentration at 0.5 mg mL^-1^. Incubation was carried out for 30 s, stopped by adding SDS-PAGE loading buffer, and heating at 100℃ for 10 min.

(b)BSA, CdDHN4-L and CdDHN4-S were treated with proteinase K. The ratio of proteinase concentration (mg mL^-1^) was varied from 1:1000 (dehydrin protein: proteinase K) to 1: 50 relative to substrate concentration at 0.5 mg mL^-1^. Incubation was carried out for 30 s, stopped by adding SDS-PAGE loading buffer, and heating at 100℃ for 10 min.
