# Supplementary material for: Characterization of Dehydrin protein, CdDHN4-L and CdDHN4-S, and their differential protective roles against abiotic stress in vitro
Source: BMC Plant Biol. 2018 Nov 26;18:299. doi: 10.1186/s12870-018-1511-2 (PMC6258397; doi:10.1186/s12870-018-1511-2)
Supplement: Supplementary file 4 — Primer sets used in this study. (DOCX 17 kb) [file 12870_2018_1511_MOESM4_ESM.docx]

**Additional file 4**

Table 2 Band positions and individual contributions by various secondary structures of CdDHN4-L and CdDHN4-S proteins determined by curve fitting of the composite amide-I band of FTIR spectra

| **Structures** | | **CdDHN4-L** | | | **CdDHN4-S** | |  | |
| --- | --- | --- | --- | --- | --- | --- | --- | --- |
| **Assignment** | **Amide-I(cm^-1^)** | | **Area(%)** | **Amide-I(cm^-1^)** | **area(%)** | **structures** | **%(L/S)** |  |
| \| Extend β-sheet \| \| --- \| | | 1633 | 21.1 | 1634 | 23.6 | Unstructured | (63.2/62.3)^a^ |  |
| random coil | | 1645 | 23.2 | 1645 | 25.7 | α-helix | (11.1/10.4) |  |
| turn+α-helix | | 1656 | 22.1 | 1655 | 20.8 | β-sheet | (25.8/27.3) |  |
| turn | | 1667 | 17.5 | 1666 | 15.9 |  |  |  |
| β-turn | | 1678 | 11.4 | 1678 | 10.3 |  |  |  |
| Extend β-sheet | | 1693 | 4.7 | 1691 | 3.7 |  |  |  |

^a^ The bands at 1,655 cm ^-1^ for both proteins in solution could be contributed by a turn and α -helix. The turn and helical structures contributed equally to the band at 1,655 cm ^-1^. Bands position and assignment of structures were according to Nevskaya and Chirgadze (1976), Byler and Susi (1986) and Shih *et al*(2010).

**References**

Byler, D. M., & Susi, H. (1986). Examination of the secondary structure of proteins by deconvolved FTIR spectra. Biopolymers, 25(3), 469-487.

Nevskaya, N. A., & Chirgadze, Y. N. (1976). Infrared spectra and resonance interactions of amide-I and II vibrations of α-helix. Biopolymers, 15(4), 637-648.

Shih, M. D., Hsieh, T. Y., Lin, T. P., Hsing, Y. I. C., & Hoekstra, F. A. (2010). Characterization of two soybean (*Glycine max* L.) LEA IV proteins by circular dichroism and Fourier transform infrared spectrometry. Plant and cell physiology, 51(3), 395-407.
